# The Oral–Gut–Systemic Axis in Periodontitis: Microbiome Dysbiosis, Immune Crosstalk and Translational Perspectives

**DOI:** 10.3390/ijms27209060

**Published:** 2026-10-13

**Authors:** Martyna Mochol, Hanna Kulig, Aleksandra Rączka, Mariusz Lipski, Ewa Marek, Laurentia Schuster, Till Dammaschke, Małgorzata Mazurek-Mochol

**Affiliations:** 1 Department of Periodontology, Pomeranian Medical University in Szczecin, Powst. Wlkp. 72, 70-111 Szczecin, Poland; 2 Department of Genetics, West Pomeranian University of Technology in Szczecin, Aleja Piastów 45, 70-311 Szczecin, Poland; 3 Department of Preclinical Conservative Dentistry and Preclinical Endodontics, Pomeranian Medical University in Szczecin, Powst. Wlkp. 72, 70-111 Szczecin, Poland; 4 Department of Periodontology and Operative Dentistry, University of Münster, 48149 Münster, Germany

**Keywords:** periodontitis, periodontal dysbiosis, oral–gut axis, gut microbiome, oral microbiota, salivary microbiota, intestinal barrier, immune crosstalk, microbial metabolites, inflammatory bowel disease, MASLD, NAFLD

## Abstract

Periodontitis is a chronic inflammatory disease associated with subgingival dysbiosis and altered host response. Although it is usually considered a local disease of the tooth-supporting tissues, it may also interact with distant mucosal and systemic compartments. One possible explanation is the oral–gut–systemic axis. In this model, oral bacteria, microbial products and inflammatory mediators are swallowed and reach the gastrointestinal tract. In susceptible hosts, these signals may affect gut microbial ecology, epithelial barrier integrity, mucosal immunity and microbial metabolite pathways. Human studies show links between periodontitis, altered oral and gut microbial communities, oral–gut microbial signatures and systemic metabolomic changes. Experimental studies provide stronger mechanistic support. They suggest that periodontal pathogens, particularly *Porphyromonas gingivalis*, may promote gut dysbiosis, barrier dysfunction, endotoxemia, inflammasome activation, T helper 17/regulatory T cell (Th17/Treg) imbalance and changes in metabolite signaling pathways. Candidate pathways include aryl hydrocarbon receptor (AHR) signaling, suppression of G protein-coupled receptor 109A (GPR109A), short-chain fatty acid-related pathways and trimethylamine N-oxide (TMAO) metabolism. Inflammatory bowel disease is currently the most developed disease-specific model. Metabolic dysfunction-associated steatotic liver disease (MASLD), non-alcoholic fatty liver disease (NAFLD), rheumatoid arthritis, cardiometabolic metabolite pathways and gastrointestinal cancer models are less mature extensions. This review summarizes current evidence and identifies the need for longitudinal studies, strain-level source tracking, multi-omics, immune phenotyping and disease-specific intervention studies. At present, the concept is biologically plausible, but clinical translation remains premature.

## 1. Introduction

Periodontitis is a chronic inflammatory disease characterized by dysbiosis of the subgingival biofilm and an altered host immune response. Newer models of periodontal pathogenesis are not centered on a single pathogen. They describe periodontitis as a polymicrobial and host-mediated disease. In this model, microbial imbalance, immune dysregulation and tissue destruction reinforce each other. Thus, periodontal dysbiosis is not merely a local microbial alteration. It is an abnormal host–microbe interaction that may increase both local and systemic inflammatory burden [1,2,3,4].

Traditionally, the systemic effects of periodontitis have been attributed to bacteremia, endotoxemia and circulating inflammatory mediators. These mechanisms remain relevant. However, they may not fully explain the relationship between periodontal disease and systemic inflammatory conditions. In recent years, the oral–gut axis has become an additional concept of interest. This axis suggests that oral dysbiosis may influence the gut microbiome, gut barrier function and mucosal immune responses. These gastrointestinal changes may then contribute to systemic metabolic and immune effects [5,6,7,8,9,10].

The oral cavity and the gastrointestinal tract are anatomically continuous. Large amounts of saliva are swallowed every day. This saliva contains oral bacteria, bacterial products, extracellular vesicles, enzymes and inflammatory mediators. Several barriers in the gut limit colonization by oral microbes. These include gastric acid, bile acids, resident gut microbiota, epithelial barriers and mucosal immunity. However, barrier dysfunction, inflammation, medication, dysbiosis and systemic disease may decrease this resistance. In such cases, oral microbes or their components may interact with the intestinal mucosa and the gut immune system [11,12,13,14].

This may be particularly relevant in periodontitis. Dysbiotic microbial communities, oral pathobionts, lipopolysaccharide (LPS), outer membrane vesicles (OMVs), proteases and inflammatory mediators are released from inflamed periodontal pockets into saliva. These signals are repeatedly swallowed. They may pass through the gut without permanent colonization, or they may persist under permissive conditions. In both cases, they may affect gut microbial ecology, epithelial barrier integrity and mucosal immune activation. A few human studies have found alterations in oral and gut microbiomes in patients with periodontitis. Some studies also indicate systemic metabolomic differences [15,16,17,18,19,20]. These results support the oral–gut hypothesis, but they do not establish directionality or causality.

For clarity, the periodontitis-related aspects considered in this review include the following: subgingival dysbiosis and pathobiont enrichment; inflamed periodontal pockets as a mucosal source of bacterial products; salivary transfer of oral bacteria, LPS, OMVs and inflammatory mediators; host inflammatory pathways involving interleukin-1β (IL-1β), interleukin-6 (IL-6), tumor necrosis factor-α (TNF-α), interleukin-17/T helper 17 (IL-17/Th17) responses, regulatory T cell (Treg) imbalance, inflammasome activation and matrix-degrading enzymes; and systemic susceptibility factors such as diabetes, obesity, smoking, medication exposure and host genetic predisposition. These aspects provide the periodontal entry points for the oral–gut–systemic axis discussed below [1,2,3,4,5,17,21,22].

Experimental studies offer stronger mechanism-based support. Several periodontal pathogens, primarily *Porphyromonas gingivalis *(*P. gingivalis)*, have been shown to shift the gut microbiome, disrupt intestinal barrier function and induce inflammatory or metabolic changes in animal models [23,24,25,26]. Other studies suggest that oral pathobionts can activate mucosal immune pathways after reaching the gut [21]. Oral inflammation may also relate to intestinal immune activation via Th17 cells, gut-homing immune responses and Th17/Treg imbalance [21,22]. More recent studies have associated periodontitis with microbial metabolite pathways, including tryptophan-aryl hydrocarbon receptor (AHR signaling), linoleic acid-AHR signaling, short-chain fatty acid (SCFA) -related signaling, G protein-coupled receptor 109A (GPR109A) suppression and trimethylamine N-oxide (TMAO) metabolism [27,28,29,30].

The strength of evidence varies across systemic disease models. Inflammatory bowel disease (IBD) is currently the strongest disease-specific model because it involves the gut microbiome, mucosal immunity and intestinal barrier function. Human and experimental studies show a relationship between periodontitis and IBD, but causality has not been established [31,32,33,34,35,36,37,38]. Other links are less developed. These include metabolic dysfunction-associated steatotic liver disease (MASLD), non-alcoholic fatty liver disease (NAFLD), rheumatoid arthritis, TMAO-related cardiometabolic pathways and gastrointestinal cancer models [27,28,39,40,41,42,43]. These conditions should not be presented as a single systemic effect of periodontitis. They should be treated as separate disease axes with different levels of evidence.

**Figure 1:**
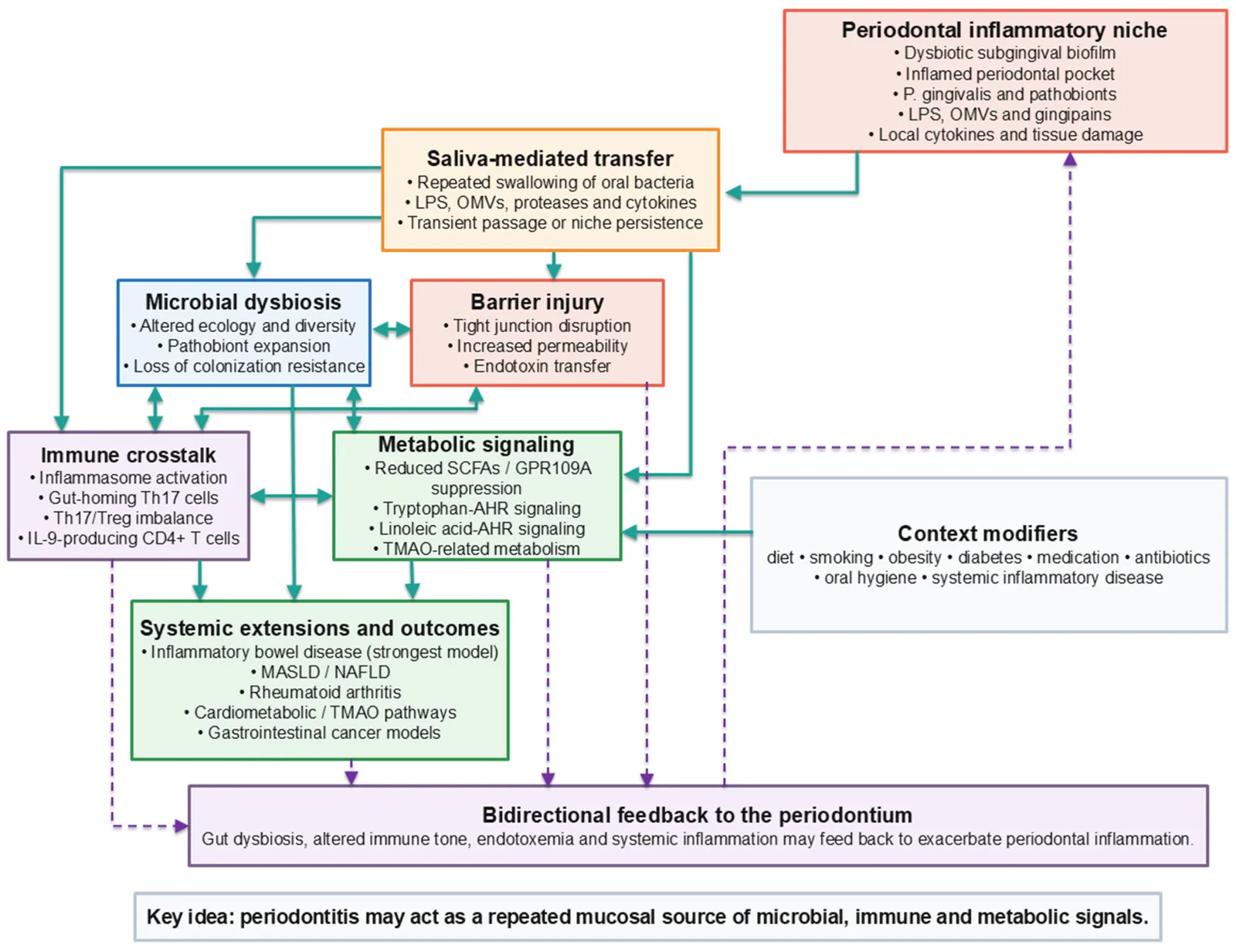
Bidirectional oral–gut–systemic signaling in periodontitis. Periodontal dysbiosis may act as a chronic mucosal source of microbial, inflammatory and metabolic signals. Swallowed saliva may transfer oral bacteria, microbial products and inflammatory mediators to the gastrointestinal tract. In susceptible hosts, these signals may contribute to gut dysbiosis, epithelial barrier injury, mucosal immune activation and systemic immune–metabolic effects. The axis is likely bidirectional, because gut dysbiosis and systemic inflammation may also influence periodontal inflammation. The strength of evidence differs between disease models. Abbreviations: AHR, aryl hydrocarbon receptor; GPR109A, G protein-coupled receptor 109A; LPS, lipopolysaccharide; MASLD, metabolic dysfunction-associated steatotic liver disease; NAFLD, non-alcoholic fatty liver disease; OMVs, outer membrane vesicles; SCFAs, short-chain fatty acids; Th17, T helper 17; TMAO, trimethylamine N-oxide; Treg, regulatory T cell. Solid arrows indicate proposed or experimentally supported directional pathways, whereas dashed arrows indicate indirect or feedback relationships. Bidirectional arrows represent reciprocal crosstalk between biological processes.

This review compiles current data on the oral–gut–systemic axis in periodontitis. It emphasizes human data, experimental mechanisms, disease-specific evidence, translational maturity and priorities for future studies. The overall concept is shown in Figure 1.

## 2. Methods

The article was prepared as a structured narrative review. Selected Preferred Reporting Items for Systematic Reviews and Meta-Analyses (PRISMA) 2020 principles were used to guide screening and reporting. The review summarizes available research on the oral–gut–systemic axis in periodontitis. The main focus was periodontal dysbiosis, oral-to-gut microbial transfer, gut microbiome disruption, intestinal barrier dysfunction, immune crosstalk, microbial metabolites and disease-specific systemic effects. Because the available literature was highly heterogeneous, a quantitative meta-analysis was not performed.

A literature search was performed in PubMed/MEDLINE, PubMed Central, Scopus, Web of Science, Embase and the Cochrane Library. Google Scholar and reference lists of relevant articles were also screened to identify additional studies. Articles published between January 2010 and May 2026 were searched. Earlier articles were included only when they were relevant to the conceptual or mechanistic background of the review.

Periodontal, oral microbiome, gut microbiome, immune and systemic disease terms were used in the search strategy. The main search terms involved were the following: periodontitis, periodontal disease, periodontal dysbiosis, periodontal inflammation, oral microbiota, salivary microbiota, periodontal microbiome, oral pathobionts, gut microbiota, intestinal microbiota, periodontal gut microbiome, oral–gut axis, gum–gut axis, oral–gut–systemic axis, dysbiosis, microbial translocation, intestinal barrier, endotoxemia, epithelial permeability, Th17, Treg, inflammasome, AHR, GPR109A, SCFA, Tryptophan, TMAO, inflammatory bowel disease, Crohn’s disease, ulcerative colitis, MASLD, NAFLD, rheumatoid arthritis, colorectal cancer and cardiometabolic disease. The terms used in the search were customized for each database.

Studies were eligible if they included periodontitis, periodontal dysbiosis or periodontal pathogens together with at least one gut-related or systemic aspect. These components included changes in gut microbiota composition, oral-to-gut microbial transfer, salivary microbiota, intestinal dysbiosis, gut barrier dysfunction, microbial metabolites, endotoxemia, mucosal immune activation or gut-derived systemic inflammatory mechanisms. Human observational studies, longitudinal studies, periodontal intervention studies, animal studies, mechanistic studies, multi-omics studies, Mendelian randomization studies, systematic reviews and high-quality narrative reviews were considered.

To avoid an overly narrow selection, eligibility was not restricted to one systemic disease, one microbiome method or one experimental design. Human observational studies, longitudinal studies, periodontal intervention studies, animal models, in vitro or biochemical studies, Mendelian randomization analyses and mechanistic reviews were considered when they directly informed oral–gut microbial transfer, host inflammation, microbial metabolites or disease-specific systemic pathways. Studies were prioritized when they clarified mechanisms relevant to periodontitis rather than only reporting generic microbiome differences.

Articles were excluded if they focused only on dental caries, peri-implantitis without periodontitis, oral microbiota without gut or systemic relevance, or gut microbiota without oral or periodontal relevance. Isolated case reports, conference abstracts without full text and non-peer-reviewed preprints without a published version were also excluded. Articles were rejected when the periodontal component was unclear.

Following the removal of duplicates, titles and abstracts were screened based on the question for the review. Potentially eligible full texts were then evaluated for eligibility. The following information was obtained from each of the included studies: author, year, type of study, population/experimental model, periodontal exposure/periodontal condition, sample type, analytical method, gut-related outcome, systemic disease context, and key mechanism and findings.

The results were synthesized narratively. This approach was selected because studies differed in design, population, experimental model, exposure, sampling method and outcome. Human studies were used to assess clinical relevance. Animal and mechanistic studies were used to assess biological plausibility. Evidence was interpreted separately for each disease axis, with attention to the strength and limitations of the available data.

When mechanistic evidence is discussed, the type of experimental approach is specified. The evidence base mainly includes mouse oral gavage or oral exposure models using *P. gingivalis*, ligature-induced periodontitis models, chemically or genetically induced colitis models, high-fat diet or obesity-related metabolic liver disease models, *apolipoprotein E-deficient* (*ApoE−/−*) cardiometabolic models, and murine arthritis or colorectal cancer models. In vitro and biochemical approaches are more limited and mainly inform microbial products, virulence factors and metabolite pathways.

**Figure 2:**
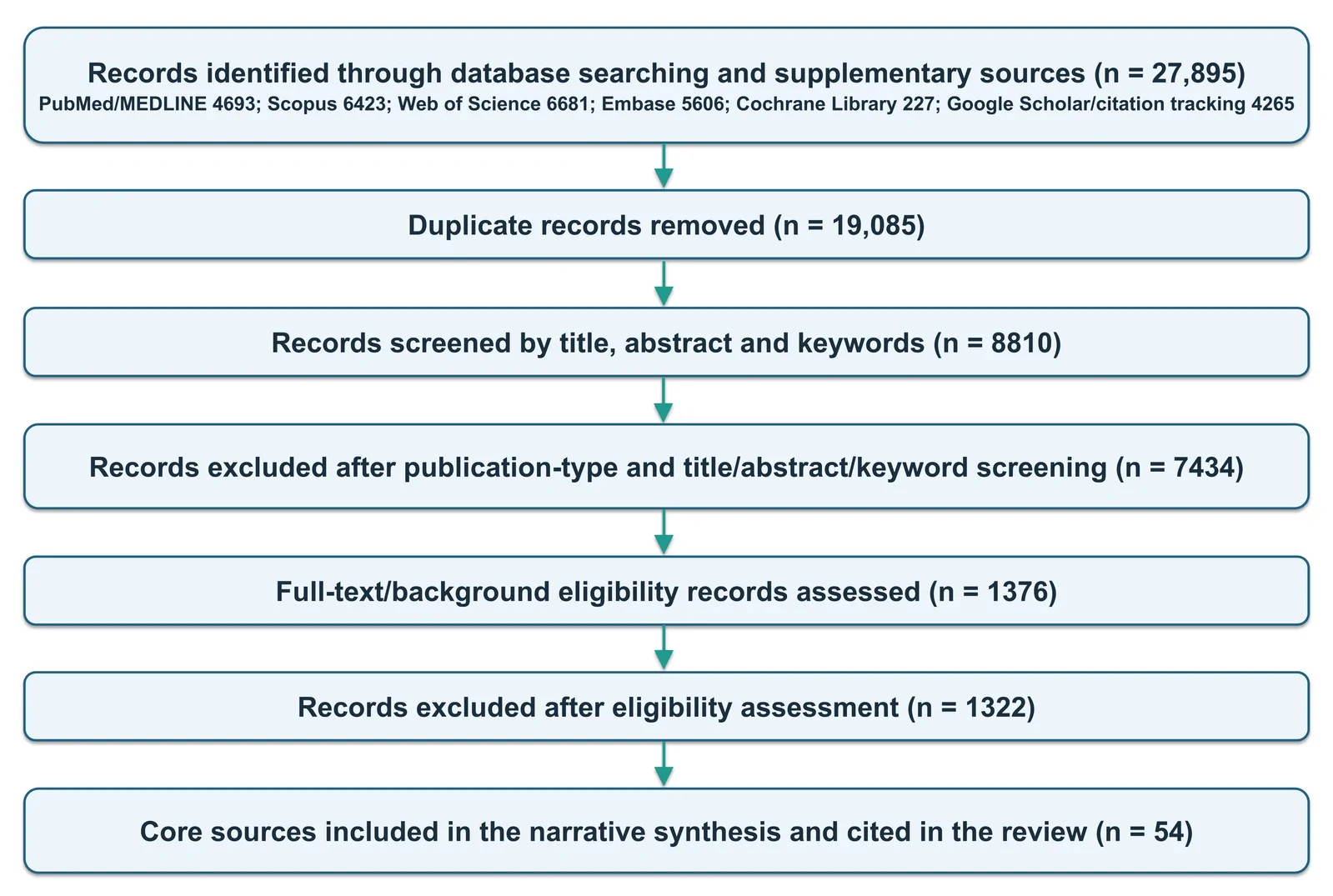
PRISMA-inspired literature selection flow diagram for the structured narrative review.

The search and selection process is summarized in Figure 2. Records identified through Cochrane Library, Google Scholar and citation tracking were treated as supplementary records and considered during duplicate removal, because these sources largely overlapped with the main database results.

## 3. Periodontal Dysbiosis as a Source of Oral–Gut–Systemic Signaling

Periodontitis begins with a disturbed relationship between the subgingival biofilm and the host immune response. In health, the periodontal microbiome is usually compatible with local tissue homeostasis. This balance is disrupted when microbial communities shift toward dysbiosis. The host response also changes. Inflammation becomes chronic and tissue damage progresses.

The polymicrobial synergy and dysbiosis model offers the best explanation of this process. In this model, periodontitis is not caused by one pathogen alone. It is mediated by a dysbiotic microbial community that modifies the local environment and regulates host immunity [1,2,3,4]. Keystone pathogens such as *Porphyromonas gingivalis* may have a strong effect even when they are present in low numbers. They can disrupt immune control and contribute to an inflammatory microbial community [1,2,3].

The periodontal pocket is more than a local ecological niche. It is also an inflamed mucosal interface. In active periodontitis, the pocket epithelium is damaged and becomes more permeable. The local inflammatory infiltrate is persistent. Bacterial products, enzymes and inflammatory mediators may be released into saliva or enter the bloodstream. In this way, the periodontal pocket may become a source of repeated microbial and inflammatory exposure.

In this context, the oral–gut connection is important. The oral cavity and the gastrointestinal tract are continuous. Saliva is swallowed throughout the day. It contains oral bacteria, bacterial products, extracellular vesicles, enzymes and host inflammatory mediators. In patients with periodontitis, saliva may contain a higher load of dysbiotic oral microorganisms and periodontal inflammatory products [17,19].

The gut is usually resistant to stable colonization by oral bacteria. Several mechanisms help maintain the integrity of the gut microbiome. These include gastric acid, bile acids, resident gut microbiota, mucus, epithelial barriers and mucosal immune responses. Together, these mechanisms provide colonization resistance and limit the long-term persistence of many oral microorganisms in the intestine.

However, this resistance is not absolute. It can be weakened by disease, diet, medication, antibiotics, inflammation, immune dysfunction or barrier damage. Under these conditions, oral bacteria and their products may have a greater effect on the gut environment. They may pass through the gut as transient signals, or they may persist in selected ecological niches when conditions allow it [11,13,14].

This distinction is important. The detection of oral bacteria in the gut does not always mean stable colonization. These bacteria may be detected but not become permanent members of the gut microbiome. Transient exposure may still be biologically relevant. During gastrointestinal passage, bacteria, lipopolysaccharide, outer membrane vesicles and proteases may come into contact with epithelial cells or immune cells.

Stable persistence may have stronger effects. If oral pathobionts persist and proliferate in the gut, they may affect microbial networks and colonization resistance. They may also alter microbial metabolism and local inflammatory status. This could contribute to intestinal dysbiosis and low-grade inflammation. However, this process is likely to occur only in susceptible hosts and permissive conditions.

The gut is thus a compartment through which periodontitis may exert several non-mutually exclusive effects. First, swallowed oral bacteria may directly interact with the intestinal mucosa. Second, bacterial products such as LPS, OMVs and proteases may influence epithelial and immune responses. Third, periodontal inflammation may contribute to systemic inflammation. Fourth, oral dysbiosis may interact with shared risk factors such as smoking, diabetes, obesity, diet and medication.

The relationship may also be bidirectional. Systemic inflammation and gut dysbiosis may affect immune regulation at distant mucosal surfaces, including the periodontium. A disturbed gut microbiome may contribute to broader immune imbalance. This could alter host susceptibility to periodontal inflammation. The oral–gut–systemic axis should therefore not be viewed as a one-way route from the mouth to the gut.

Human studies support the idea that periodontitis may be associated with systemic changes. Severe periodontitis has been linked with disturbances in salivary and fecal microbiota [17]. Combined oral and gut profiles may also help distinguish periodontal health from disease [19]. Periodontal therapy has been linked to shifts in the composition of oral and gut microbiota [15]. These findings support the idea of oral–gut interaction, but they do not establish a cause-and-effect relationship.

This conceptual framework is presented in Figure 1. Detailed human evidence and experimental mechanisms are discussed in Section 4 and Section 5.

## 4. Human Evidence for Oral–Gut Microbiome Disruption in Periodontitis

Human evidence for the oral–gut axis in periodontitis has increased in recent years. These studies suggest that periodontitis may be associated with changes in the gut microbiome and systemic metabolic profile. However, the evidence is still less mature than the experimental evidence. Most human studies are small, exploratory or observational. They can show associations, but they cannot prove direct causality.

The main human studies are summarized in Table 1. They include exploratory fecal microbiome studies, paired saliva and stool studies, periodontal therapy studies, multi-omics studies and Mendelian randomization analyses. Together, they support the idea that periodontitis may be linked with changes beyond the oral cavity. At the same time, they show that the direction of this relationship is still uncertain.

Table 1 supports the aim of this review by showing which elements of the proposed oral–gut–systemic axis are currently supported by human data. The studies do not prove mechanisms, but they define the clinically relevant evidence base: paired oral and fecal microbial signatures, gut microbial differences according to periodontal status, periodontal therapy-associated changes, systemic metabolomic patterns and causal hypotheses generated by Mendelian randomization.

Early work suggested that gut microbial profiles may differ according to periodontal status [18]. This was important because it moved the field beyond the idea that periodontitis is only a local oral disease. It also showed how difficult this type of research can be. Diet, age, smoking, medication, obesity, diabetes and systemic inflammation can all affect the gut microbiome. Many of these factors are also linked with periodontal disease. This makes causal interpretation difficult.

Paired saliva and fecal microbiome studies provide more direct support for the oral–gut concept. Bao et al. compared salivary and fecal microbiota in patients with severe periodontitis and periodontally healthy controls [17]. The study showed that severe periodontitis was associated with changes in both oral and gut microbial communities. This supports the idea that dysbiotic salivary microbiota may contribute to gut microbial disturbance. However, the study does not prove that oral bacteria permanently colonize the gut. It also does not prove that gut dysbiosis is caused by periodontitis.

Other human studies suggest that combined oral and gut microbial profiles may help distinguish periodontal health from disease [19]. This is important from a diagnostic and translational point of view. It suggests that periodontitis may have a broader microbial signature. This signature may include both oral and intestinal compartments. However, these profiles should be treated as associative biomarkers. They are not ready for clinical use.

Interventional studies are especially important because they can test whether periodontal treatment is followed by changes in the gut microbiome. Baima et al. studied the effect of periodontitis and periodontal therapy on oral and gut microbiota [15]. Periodontal treatment was associated with changes in gut microbial profiles. Some changes appeared to move toward a healthier pattern. This suggests that local periodontal treatment may have extraoral microbial effects.

This interpretation must remain cautious. Periodontal treatment changes many things at the same time. It reduces oral inflammation. It changes plaque control. It may change oral hygiene behavior, antiseptic use, diet, medication exposure and general health behavior. All these factors may also influence the gut microbiome. Therefore, treatment-associated gut microbial changes should not be interpreted as proof of a direct oral-to-gut causal pathway.

More recent studies have added metabolomic and multi-omics data. Miyauchi et al. reported persistent gut dysbiosis and a distinct serum metabolome in patients with periodontitis [20]. This is important because it links periodontitis not only with microbial composition, but also with systemic metabolic signatures. The reported reduction in short-chain fatty acid-producing bacteria may be relevant to immune and metabolic regulation. However, persistent gut dysbiosis after periodontal therapy also suggests another possibility. Gut disruption may not be only a reversible consequence of local periodontal inflammation. It may reflect a broader host–microbiome imbalance.

Baima et al. also used a multi-omics approach to study periodontitis and periodontal therapy across oral and gut compartments [16]. This type of study is important because taxonomy alone is not enough. Knowing which bacteria are present does not show what they are doing. Functional and metabolic data can give more useful information. They may help identify microbial pathways linked with disease activity, treatment response or systemic risk.

Mendelian randomization studies suggest possible links between gut microbial taxa and periodontitis risk [44,45,46]. Paired oral and gut profiling in periodontitis and Parkinson’s disease further shows the value of combined sampling in complex systemic disease contexts [47].

## 5. Experimental Mechanisms: Oral Pathobionts, Barrier Dysfunction and Immune–Metabolic Crosstalk

**Figure 3:**
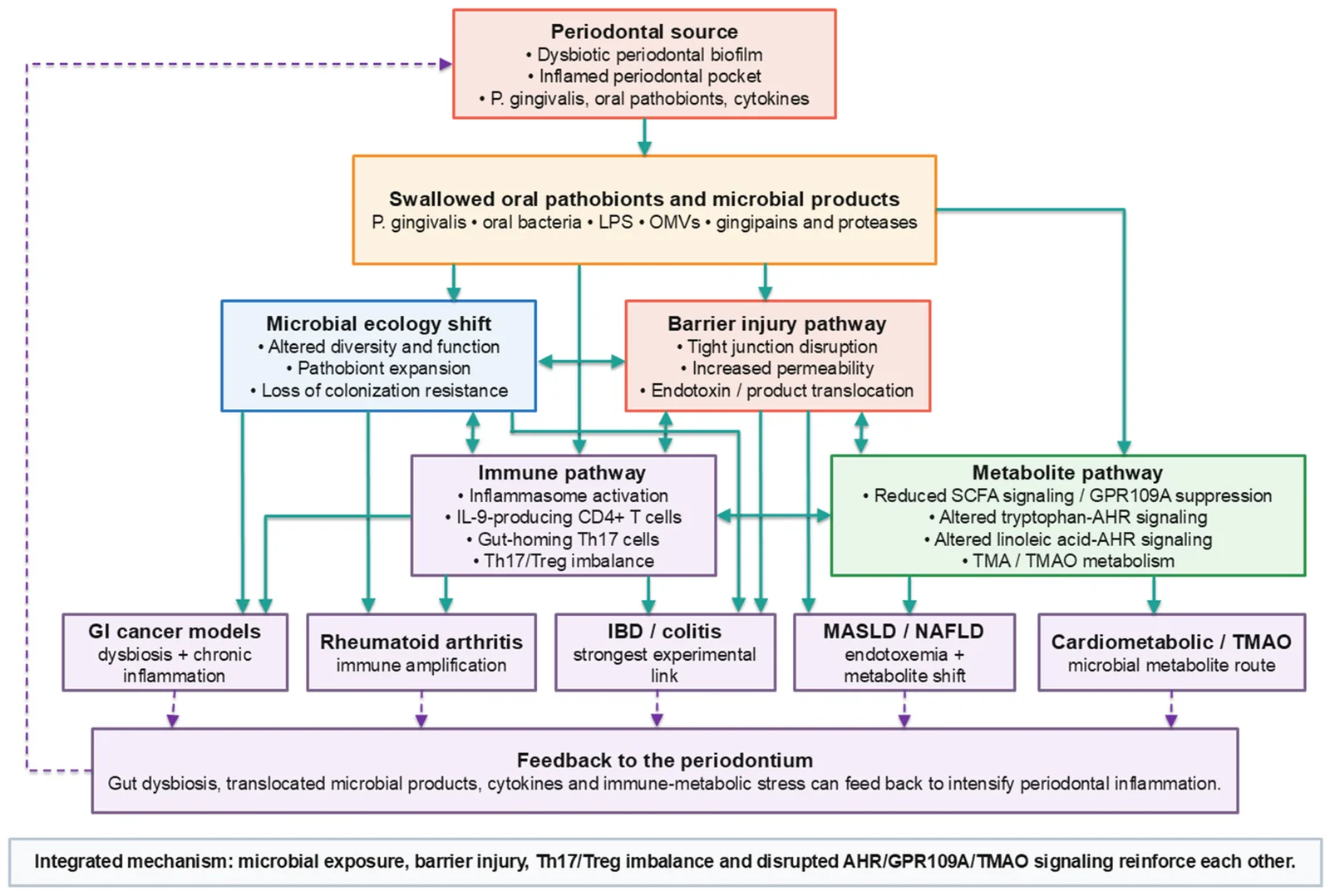
Molecular and immunological pathways linking periodontal dysbiosis with gut and systemic inflammation. Swallowed oral pathobionts and microbial products may affect the gut through several interacting pathways. These include gut microbial dysbiosis, epithelial barrier dysfunction, inflammasome activation, IL-9-producing CD4+ T-cell responses, gut-homing Th17 cells, Th17/Treg imbalance and microbial metabolite disruption. Metabolic pathways include reduced SCFA-GPR109A signaling, altered tryptophan-AHR and linoleic acid-AHR signaling, and TMAO-related metabolism. These mechanisms provide biological links between periodontitis, intestinal inflammation and systemic immune–metabolic disease models, but their clinical relevance requires further human validation. Abbreviations: AHR, aryl hydrocarbon receptor; CD4, cluster of differentiation 4; GI, gastrointestinal; GPR109A, G protein-coupled receptor 109A; IBD, inflammatory bowel disease; LPS, lipopolysaccharide; MASLD, metabolic dysfunction-associated steatotic liver disease; NAFLD, non-alcoholic fatty liver disease; OMVs, outer membrane vesicles; SCFA, short-chain fatty acid; TMA, trimethylamine; TMAO, trimethylamine N-oxide; Th17, T helper 17; Treg, regulatory T cell. Solid arrows represent proposed or experimentally supported mechanistic pathways and crosstalk. Dashed arrows indicate indirect systemic feedback to the periodontium. Bidirectional arrows indicate reciprocal interactions between microbial, barrier, immune and metabolic pathways.

Experimental studies provide the strongest mechanistic support for the oral–gut–systemic axis in periodontitis. They test how oral pathobionts and their products may affect the gut microbiome, epithelial barrier, mucosal immunity and systemic metabolism. The main pathways are summarized in Table 2 and Figure 3.

**Table 2:** Experimental mechanisms supporting the oral–gut–systemic axis in periodontitis.

Pathway	Key Study/Experimental Approach	Molecular or Immune Mechanism	Systemic Implication	Main Limitation
Oral pathobiont exposure and gut dysbiosis	Nakajima et al. [24]; oral *P. gingivalis* administration in mice	Oral administration of *P. gingivalis* altered gut microbiota and impaired gut barrier function.	Supports the idea that oral periodontal pathogens can disturb intestinal homeostasis.	Oral gavage models may not fully reflect chronic human periodontitis.
Microbiome–metabolome interaction	Kato et al. [23]; Dong et al. [48]; oral *P. gingivalis* exposure with gut microbiome and serum metabolome profiling	*P. gingivalis* exposure changed the gut microbiome and serum metabolome.	Links oral dysbiosis with systemic metabolic effects.	Metabolic changes need stronger disease-specific validation in humans.
Gingipain-dependent intestinal inflammation	Li et al. [49]; experimental *P. gingivalis* exposure and gingipain-dependent intestinal inflammation model	*P. gingivalis* induced gut dysbiosis and intestinal inflammation through gingipain-dependent mechanisms.	Suggests that bacterial virulence factors may drive gut inflammation.	Evidence is experimental and requires clinical confirmation.
Barrier dysfunction and endotoxin transfer	Nakajima et al. [24]; Tsuzuno et al. [26]; oral *P. gingivalis* exposure and mouse intestinal barrier/colitis models	Oral *P. gingivalis* exposure disrupted intestinal epithelial barrier integrity and promoted inflammatory responses.	Provides a route from oral dysbiosis to intestinal and systemic inflammation.	Barrier injury is a broad mechanism and may not be disease specific.
IL-9 and CD4+ T-cell pathway	Sohn et al. [25]; *P. gingivalis* exposure model with CD4+ T-cell responses	*P. gingivalis* indirectly promoted intestinal inflammation through microbiota changes and IL-9-producing CD4+ T cells.	Shows that oral pathobionts may act through host immune pathways, not only through colonization.	The role of this pathway in human periodontitis remains unclear.
Inflammasome and Th17 cell trafficking	Kitamoto et al. [21]; pathobiont-driven experimental colitis mouse model with gut-homing T cells	Oral pathobionts promoted colitis through inflammasome activation and gut-homing Th17 cells.	Provides one of the strongest models of mouth–gut immune crosstalk.	The model is based on experimental colitis-prone conditions and may not directly reproduce human disease.
Gut–mouth Th17 immune loop	Nagao et al. [22]; mouse gut–mouth Th17 immune axis model	Pathobiont-responsive Th17 cells linked gut dysbiosis with inflammatory oral disease.	Supports bidirectional mucosal immune crosstalk between gut and mouth.	The pathway is biologically important but not yet clinically measurable in routine practice.
Linoleic acid-AHR-Th17/Treg pathway	Jia et al. [29]; *P. gingivalis*-exacerbated experimental colitis mouse model	*P. gingivalis* aggravated colitis through gut microbiota changes, linoleic acid metabolism, AHR signaling and Th17/Treg imbalance.	Connects periodontal dysbiosis with metabolite-driven immune imbalance.	The findings need validation outside experimental colitis models.
SCFA-GPR109A pathway	Wang et al. [30]; mouse periodontitis-associated intestinal inflammation model	Periodontitis promoted intestinal inflammation through gut microbiota-dependent suppression of GPR109A.	Suggests that loss of protective SCFA signaling may contribute to gut inflammation.	Human relevance and therapeutic value remain uncertain.
Tryptophan-AHR pathway	Chen et al. [27]; obesity/metabolic liver disease mouse model	Periodontitis worsened metabolic liver disease through gut microbiota-derived tryptophan metabolism and AHR signaling.	Links oral dysbiosis with the oral–gut–liver axis.	The pathway is supported mainly by obesity and metabolic disease models.
TMAO-related metabolism	Huang et al. [28]; ApoE−/− mouse model with periodontitis and periodontal treatment	Nonsurgical periodontal treatment improved abnormal TMAO metabolism in ApoE−/− mice with periodontitis.	Suggests a possible link between periodontal therapy, gut metabolites and cardiometabolic risk.	ApoE−/− mouse findings may not predict cardiovascular outcomes or TMAO changes in humans.
Experimental therapeutic modulation	Huang et al. [50]; ApoE−/− periodontitis model; Qu et al. [51]; microbiota-modulation model of *P. gingivalis*-exacerbated intestinal inflammation	Periodontal treatment or microbiota-modulating agents improved gut barrier or inflammatory outcomes in experimental models.	Supports the idea that oral or gut-directed interventions may affect systemic pathways.	These approaches remain investigational and should not be presented as established therapy.

Table 2 and Figure 3 complement the text by separating broad concepts from testable pathways. Table 2 identifies the experimental approach behind each line of evidence, while Figure 3 visually connects oral pathobiont exposure with barrier injury, immune activation, Th17/Treg imbalance, AHR-related signaling, SCFA-GPR109A signaling and TMAO-related metabolism. This organization links the experimental evidence directly to the aim of the review.

### 5.1. Oral Pathobiont Exposure and Gut Dysbiosis

Oral exposure to *Porphyromonas gingivalis* is one of the main experimental models in this field. *P. gingivalis* is often described as a keystone periodontal pathogen. It may disturb host immune control and reshape microbial communities even when it is not highly abundant [1,2,3].

In experimental models, oral *P. gingivalis* exposure can alter the gut microbiome. Nakajima et al. showed that oral administration of *P. gingivalis* induced gut dysbiosis and impaired intestinal barrier function. This was followed by dissemination of *Enterobacteriaceae* to the liver [24]. This study is important because it connects oral pathogen exposure with gut microbial disruption, barrier damage and extraintestinal effects.

Kato et al. extended this concept by showing that oral *P. gingivalis* exposure changed both the gut microbiome and the serum metabolome [23]. Dong et al. also linked *P. gingivalis*-related metabolic disturbance with changes in gut microbiota and serum metabolites [48]. Gingipain-dependent mechanisms may also contribute to intestinal inflammation after *P. gingivalis* exposure [49]. Together, these studies suggest that oral pathobiont exposure may have effects beyond local gut dysbiosis. It may also influence systemic metabolic profiles.

### 5.2. Barrier Dysfunction and Microbial Product Translocation

Intestinal barrier dysfunction is one of the main mechanisms linking oral pathobionts with gut and systemic inflammation. The gut barrier limits contact between luminal microbes and host tissues. When this barrier is weakened, bacterial products may interact more strongly with epithelial and immune cells.

Several experimental studies support this mechanism. Oral *P. gingivalis* exposure has been linked with impaired intestinal epithelial barrier integrity [24,26]. This may allow microbial products, endotoxin or bacteria-derived signals to reach immune cells and extraintestinal tissues.

### 5.3. Immune Crosstalk: IL-9, Inflammasome Activation and Th17 Responses

Experimental work also shows that oral pathobionts may affect the gut through immune pathways. This is important because oral bacteria do not need to become stable gut colonizers to have biological effects. They may change the resident gut microbiota, activate epithelial responses or trigger immune signaling.

Sohn et al. reported that *P. gingivalis* indirectly induced intestinal inflammation by altering the gut microbiota and disrupting epithelial barrier function through IL-9-producing cluster of differentiation 4-positive (CD4+) T cells [25]. This model shows that the effect of oral pathobionts may be indirect. The main pathway may involve resident gut microbes and host immune cells, not simple pathogen colonization.

Kitamoto et al. provided one of the strongest models of mouth–gut immune crosstalk. In their model, oral pathobionts promoted colitis through an intermucosal mechanism involving inflammasome activation and gut-homing Th17 cells [21]. This study is important because it links oral microbial exposure with immune cell trafficking and intestinal inflammation.

Nagao et al. added another layer to this concept. They showed that pathobiont-responsive Th17 cells in the gut–mouth axis can provoke inflammatory oral disease and are modulated by the intestinal microbiome [22]. This supports a bidirectional mucosal immune loop. The gut may not only receive oral inflammatory signals. It may also shape immune responses that affect the periodontium.

### 5.4. Microbial Metabolite Pathways: AHR, GPR109A, SCFA and TMAO

Microbial metabolites are another important part of the oral–gut–systemic axis. Gut dysbiosis may change the production of metabolites that regulate barrier function, immune balance and systemic inflammation. These pathways may help explain how periodontal dysbiosis could contribute to metabolic or immune effects beyond the oral cavity.

AHR-related metabolism is one example. Jia et al. showed that *P. gingivalis* aggravated colitis through a gut microbiota–linoleic acid metabolism–Th17/Treg cell balance axis [29]. This study connects oral pathobiont exposure with microbial metabolite change, AHR signaling and adaptive immune imbalance.

A related mechanism has been reported in metabolic liver disease. Chen et al. showed that periodontitis exacerbated metabolic dysfunction-associated steatotic liver disease through gut microbiota-derived tryptophan metabolism and AHR signaling in obesity [27]. This supports a possible oral–gut–liver pathway. It also suggests that the effect of periodontitis may be stronger in metabolically susceptible hosts.

The SCFA-GPR109A pathway is another candidate mechanism. Wang et al. reported that periodontitis promoted intestinal inflammation through gut microbiota-induced suppression of GPR109A [30]. This suggests that loss of protective microbial metabolite signaling may contribute to gut inflammation.

TMAO-related metabolism may connect periodontitis with cardiometabolic pathways. Huang et al. showed that nonsurgical periodontal treatment improved abnormal TMAO metabolism in ApoE−/− mice with periodontitis [28]. This does not prove that periodontal therapy reduces cardiovascular risk in humans. However, it supports the idea that periodontal inflammation may interact with gut microbial metabolism.

### 5.5. Experimental Therapeutic Modulation

Several experimental studies suggest that oral or gut-related pathways can be modified. Huang et al. reported that nonsurgical periodontal treatment restored parts of the gut microbiota and intestinal barrier function in ApoE−/− mice with periodontitis [50]. In another study, periodontal treatment improved abnormal TMAO metabolism in the same type of model [28]. Qu et al. also suggested that pharmacological modulation of the gut microbiota could reduce *P. gingivalis*-exacerbated intestinal inflammation [51].

These studies are useful because they show that the axis may be modifiable. However, they should not be presented as direct clinical evidence. They are preclinical studies. They do not prove that periodontal treatment or gut-targeted agents improve systemic disease outcomes in humans.

### 5.6. Summary of Experimental Evidence

Experimental evidence supports a multi-layered model of the oral–gut–systemic axis in periodontitis. Periodontal dysbiosis may expose the gut to oral pathobionts and microbial products. These signals may disturb the gut microbiome, weaken the epithelial barrier, activate mucosal immune pathways and alter microbial metabolite signaling.

The strongest experimental mechanisms include *P. gingivalis*-driven gut dysbiosis, epithelial barrier injury, IL-9-producing CD4+ T-cell responses, inflammasome activation, gut-homing Th17 cells, Th17/Treg imbalance, AHR-related metabolism, SCFA-GPR109A signaling and TMAO-related metabolism. These mechanisms are biologically plausible and increasingly detailed. However, most evidence comes from animal models, pathogen administration studies or specific disease models such as colitis, ApoE−/− mice or metabolic liver disease.

Future studies should also include host inflammatory and immune markers that are directly relevant to periodontitis. Candidate markers include IL-1β, IL-6, TNF-α, IL-17/Th17-related cytokines, C-reactive protein (CRP), neutrophil-related mediators, MMPs, anti-*P. gingivalis* antibody responses and markers of Treg imbalance. Genetic and host-response factors may also modify susceptibility to periodontal destruction and systemic inflammatory signaling. These markers should be measured together with oral and gut microbiome data, because inflammation is the biological link through which periodontal dysbiosis may influence the oral–gut–systemic axis.

## 6. Disease-Specific Axes: Molecular Mechanisms and Evidence Strength

Not all systemic areas linked with periodontitis support the oral–gut–systemic axis to the same degree. Human studies, cohort data, reviews and experimental models support some links, whereas others rely mainly on preclinical or associative evidence. Therefore, disease axes should not be treated as one uniform systemic effect of periodontitis.

**Figure 4:**
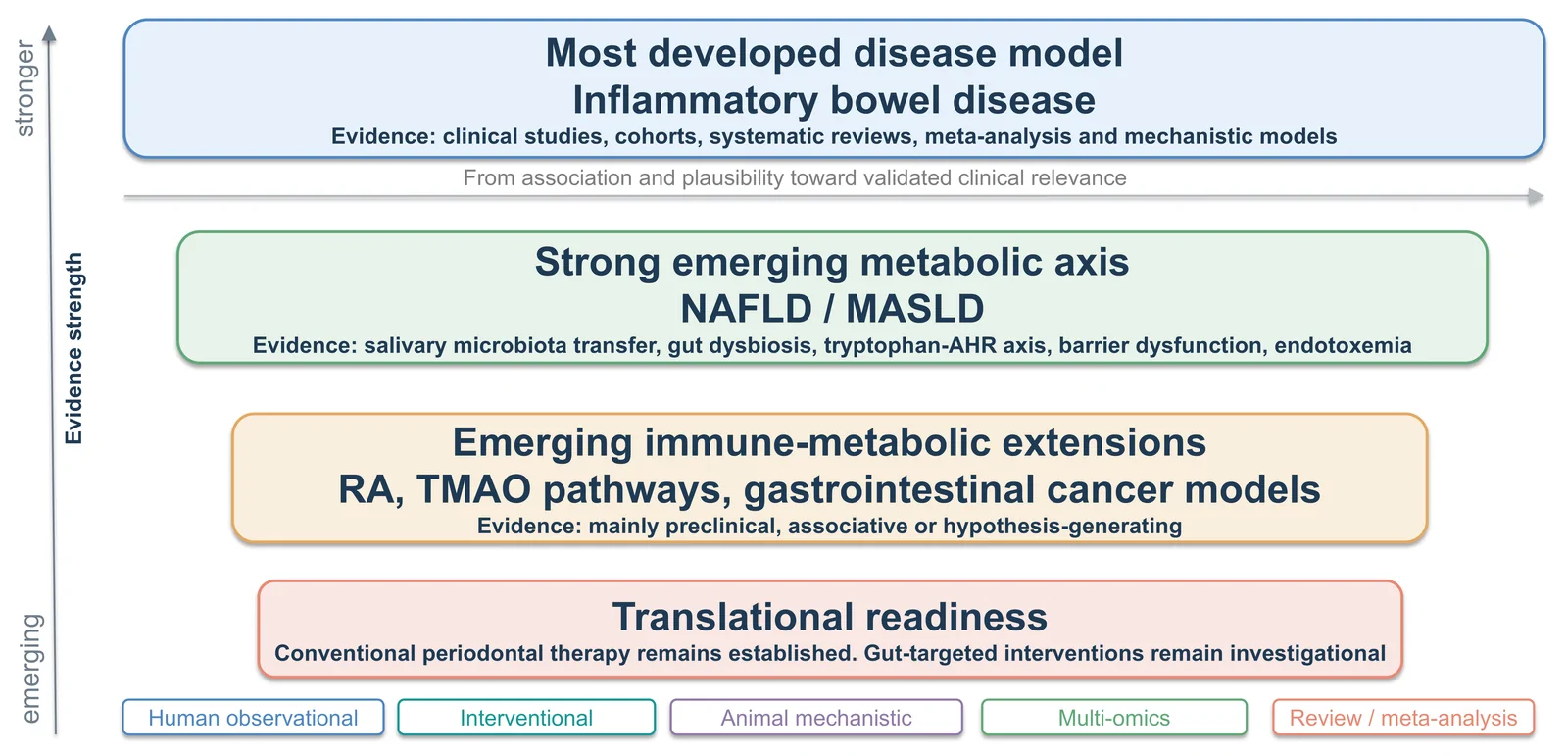
Evidence hierarchy and translational maturity of oral–gut–systemic disease axes. Disease-specific links differ in strength of evidence and readiness for clinical translation. Inflammatory bowel disease is currently the most developed disease-specific model. MASLD/NAFLD has strong mechanistic support, especially in oral–gut–liver models, but still requires stronger human validation. Rheumatoid arthritis, TMAO-related cardiometabolic pathways and gastrointestinal cancer models remain promising but less mature. The figure emphasizes that the oral–gut–systemic axis should not be treated as one uniform mechanism across all systemic diseases. Abbreviations: IBD, inflammatory bowel disease; MASLD, metabolic dysfunction-associated steatotic liver disease; NAFLD, non-alcoholic fatty liver disease; RA, rheumatoid arthritis; TMAO, trimethylamine N-oxide.

The main disease-specific mechanisms are summarized in Table 3. Inflammatory bowel disease is currently the most developed disease-specific model. Less mature extensions include MASLD/NAFLD, rheumatoid arthritis, TMAO-related cardiometabolic pathways and gastrointestinal cancer models. These axes have biological plausibility, but they require stronger human validation. The various levels of evidence are summarized in Figure 4.

### 6.1. Inflammatory Bowel Disease

The oral–gut–systemic axis is most advanced in relation to IBD. This is because IBD is directly connected to the gut microbiome, intestinal barrier function and mucosal immune regulation. These are also primary compartments of the oral–gut axis affected by oral microbial and inflammatory signals.

There are several possible links between periodontitis and IBD. Periodontal pathobionts and their products may be ingested with swallowed saliva. In susceptible hosts, they may disrupt gut microbial ecology, impair epithelial barrier function and activate mucosal immunity. Experimental research also indicates a role for inflammasome activation, gut-homing Th17 cells and Th17/Treg imbalance [21,26,29,52].

Human evidence also supports an association between periodontitis and IBD. Clinical studies, cohort data, systematic reviews and meta-analyses indicate that the relationship may be bidirectional [31,32,33,34,35,36,37,38]. However, this does not prove direct causality. Periodontal disease and systemic inflammatory disorders such as IBD can both be influenced by smoking, diet, medication, oral hygiene and health behavior.

### 6.2. MASLD/NAFLD and the Oral–Gut–Liver Axis

MASLD and NAFLD are important metabolic components of the oral–gut–systemic axis. The liver is closely connected with the gut through the portal circulation. It is therefore exposed to microbial products, metabolites and inflammatory mediators derived from the intestine.

Experimental evidence indicates that periodontitis may exacerbate metabolic liver disease in susceptible hosts. In high-fat diet or obesity models, periodontitis-associated salivary microbiota may affect the gut microbiota and contribute to liver inflammation [43]. Other evidence suggests that gut microbiota-derived tryptophan metabolism and AHR signaling may connect periodontitis with worsening MASLD [27].

This axis is mechanistically well supported, but the evidence is still largely preclinical. Obesity, insulin resistance, diet, alcohol intake, medication and metabolic syndrome are all involved in human MASLD. These factors make causal interpretation difficult.

### 6.3. Rheumatoid Arthritis

Rheumatoid arthritis (RA) is a plausible immune-mediated extension of the oral–gut–systemic axis. Periodontitis and RA share some features, including chronic immune activation, tissue-destructive inflammation and microbial dysbiosis. Previous studies have focused on *P. gingivalis*, citrullination and systemic autoimmunity. The oral–gut axis provides another possible mechanism.

Experimental studies suggest that periodontitis-associated salivary microbiota may exacerbate arthritis through gut dysbiosis and immune dysfunction [40]. This supports a possible gut-dependent mechanism, although robust human oral–gut–RA studies are still needed.

### 6.4. Cardiometabolic Pathways and TMAO

TMAO metabolism is a potential connection between the gut microbiome, systemic metabolism and cardiovascular risk. It may also be relevant to periodontitis if periodontal inflammation alters gut microbial metabolism.

Although not definitive, experimental data indicate that nonsurgical periodontal treatment may improve abnormal TMAO metabolism in ApoE−/− mice with periodontitis [28]. This does not show that periodontitis affects cardiovascular outcomes in humans through TMAO. Thus, TMAO should be considered a mechanistic candidate, not a clinical periodontal biomarker.

### 6.5. Gastrointestinal Cancer

Gastrointestinal cancer is a more hypothetical extension of the oral–gut–systemic axis. Oral microbial translocation, gut dysbiosis, immune modulation and chronic inflammation may contribute to a pro-inflammatory or pro-tumor gut environment in the context of periodontal dysbiosis [39,42].

Experimental evidence supports this possibility. In a mouse model, ligature-induced periodontitis promoted colorectal cancer and was associated with remodeling of the oral and gut microbiomes and immune suppression [41].

Cancer is a multifactorial disease. Genetics, diet, obesity, alcohol, smoking, immune status and many other environmental factors have an influence. Thus, it would be incorrect to suggest that oral–gut microbial changes are a direct causal factor in gastrointestinal cancer.

### 6.6. Summary of Disease-Specific Evidence

The strength of evidence differs across disease axes. IBD is the most developed model because it includes human associations, epidemiological data and experimental mechanisms. MASLD/NAFLD has good mechanistic support, particularly through the oral–gut–liver axis, but still needs better human validation. RA, TMAO-related pathways and gastrointestinal cancer remain less developed.

This hierarchy is important. The oral–gut–systemic axis is not a universal pathway explaining all systemic associations with periodontitis. It should be presented as a set of disease-specific mechanisms. Each mechanism has a different evidence base and a different level of clinical readiness.

## 7. Multi-Omics and Biomarker Development

The oral–gut–systemic axis in periodontitis is a complex biological process. It involves microbial communities, microbial function, epithelial barrier integrity, host immune responses and systemic metabolic changes. As a result, taxonomic microbiome profiling is insufficient. It can identify which bacteria are present, but it does not fully explain what these bacteria are doing or how they interact with the host.

Previous microbiome research in periodontitis mainly focused on bacterial abundance. This approach remains useful for comparing periodontal health and disease and for detecting oral and fecal microbial patterns associated with periodontitis [17,18,19]. However, taxonomic profiling has limitations. The presence of a bacterial taxon does not always indicate bacterial activity, virulence or metabolic output. Different strains of the same species may also have different biological effects. Future studies therefore need to move from the question “who is there?” to the questions “what are they doing?” and “how do they affect host pathways?”

Multi-omics approaches may help answer these questions. Shotgun metagenomics can identify microbial genes and functions. Metatranscriptomics can reveal which microbial genes are expressed. Metabolomics can measure microbial and host metabolites. Proteomics can provide information about host and microbial proteins. When used together, these techniques can provide a more comprehensive profile of the oral–gut–systemic axis.

Metabolomics is particularly relevant. Gut microbiota can produce or transform many metabolites that influence barrier function, immune regulation, hepatic metabolism and systemic inflammation. These include short-chain fatty acids, tryptophan metabolites, linoleic acid-related metabolites, bile acids and trimethylamine N-oxide-related metabolites. These pathways are important because they link microbial dysbiosis with host physiology.

Some human data now support this approach. Miyauchi et al. found that patients with periodontitis had persistent gut dysbiosis and a distinct serum metabolome [20]. Baima et al. showed that periodontitis and periodontal therapy were associated with changes in oral and gut microbial compartments [15,16]. These results are important, but they do not prove a direct clinical effect of oral-to-gut exposure.

Biomarker development is a logical next step. There are several biological compartments in the oral–gut–systemic axis. Saliva may reflect oral microbial burden, periodontal inflammation and dysbiotic oral communities. Fecal samples may provide information about gut microbial composition and metabolic capacity. Serum or plasma may indicate systemic inflammation and exposure to host and microbial metabolites. Combining these compartments may provide more information than one sample type alone.

Each sample type has its own limitations. Oral hygiene, food intake, medications, saliva flow and sampling protocol influence salivary biomarkers. Fecal samples mostly represent distal luminal microbiota. Diet, liver and kidney function, obesity, diabetes and medication can strongly affect serum metabolomics.

Risk stratification is one potential application of oral–gut–systemic biomarkers. Some patients may present with mainly local periodontal disease, whereas others may also show gut dysbiosis, metabolite changes or elevated systemic inflammatory markers. These signatures might also help explain biological responses after periodontal therapy.

This is a significant point to note. Periodontal staging and grading are still based mainly on clinical and radiographic findings. Examples of these are probing depth, clinical attachment loss, bleeding on probing, radiographic bone loss, tooth loss and risk factors. In the future, multi-omics biomarkers might contribute biological information to the clinical diagnosis of periodontal disease, but they cannot currently be used in place of clinical periodontal diagnosis.

The long-term goal is to determine whether oral–gut–systemic signatures can improve patient care. They may help identify patients with systemic inflammatory susceptibility. They may also help predict treatment response or identify patients who could benefit from future adjunctive interventions. Conceptual evaluations of the oral–gut axis indicate that precision periodontology may eventually assess periodontal, intestinal and systemic compartments together [6,9,10].

Currently, this is a research objective. Oral–gut biomarker thresholds have not been validated, and there is no evidence that microbiome testing enhances periodontal outcomes. Development of biomarkers should thus continue to be tied to clinically relevant endpoints.

## 8. Future Research Roadmap and Translational Priorities

The concept of the oral–gut–systemic axis in periodontitis is promising but requires further validation. Current evidence supports biological plausibility, but it does not yet provide a clear clinical pathway. The next step is to progress from broad association to pathway-specific testing.

A limitation of this review is that periodontitis is influenced by many local and systemic modifiers. Plaque control, smoking, diabetes, obesity, medication exposure, age, socioeconomic factors, oral hygiene behavior and host genetic predisposition may all affect disease occurrence, severity and response to therapy. These factors may also influence the gut microbiome and systemic inflammatory phenotype. Future precision studies should therefore combine periodontal diagnosis with host-risk profiling, inflammatory markers and, where appropriate, genetic or epigenetic susceptibility data. Gene-targeted or host-response-directed therapy remains speculative, but it may become relevant if specific pathways are validated in well-defined patient subsets.

### 8.1. Moving Beyond Association

Future studies need to go beyond simple comparisons of microbiome structure between patients with and without periodontitis. Large longitudinal cohorts should include repeated oral, salivary, fecal and systemic sampling with detailed periodontal staging and grading. Periodontal intervention studies should also include gut-related endpoints such as fecal microbiome composition, microbial metabolites, intestinal barrier markers and systemic inflammatory mediators.

### 8.2. Identifying True Oral-to-Gut Microbial Transmission

One of the main challenges is to determine whether oral bacteria found in the gut are truly transmitted from the mouth. It is not sufficient to find the same taxon in stool and saliva. The same bacterial name should not be assumed to indicate the same strain spreading from mouth to gut.

Future studies should use strain-level metagenomics, source tracking algorithms and longitudinal sampling. This is especially important for organisms such as *Porphyromonas gingivalis, Fusobacterium nucleatum*, *Klebsiella* spp. and *Enterobacter* spp. Strain-level resolution may help differentiate transient oral shedding from stable intestinal colonization.

### 8.3. Integrating Multi-Omics and Immune Phenotyping

Taxonomic profiling alone is not enough. It can indicate which microbes are present, but not what they are doing. Many important effects depend on microbial genes, virulence factors, metabolites and host immune responses.

Future studies should use multi-omics approaches, including shotgun metagenomics, meta-transcriptomics, metabolomics, proteomics and immune profiling. This would link microbial composition with microbial function. It would also allow researchers to test whether pathways observed in experimental models are detectable in humans.

Specific pathways should be explored, especially those that already have mechanistic support. These include epithelial barrier dysfunction, inflammasome activation, Th17/Treg imbalance, gut-homing T cells, tryptophan-AHR signaling, linoleic acid-AHR signaling, SCFA-GPR109A signaling and TMAO-related metabolism. These pathways should not only be described but also measured and validated in well-designed human studies.

### 8.4. Standardizing Periodontal and Microbiome Protocols

Standardization is essential. Studies should use clear periodontal terminology. They should report periodontal stage, grade, probing depth, clinical attachment loss, bleeding on probing, radiographic bone loss and treatment status. Active untreated periodontitis should not be combined with stable treated periodontitis unless this is clearly reported.

Sampling methods also need standardization. Oral samples can include subgingival plaque, supragingival plaque, saliva, tongue coating or tongue swabs. These sites are biologically different. One of the main reasons for the importance of saliva in the oral–gut axis is the fact that it is continuously swallowed. However, the subgingival biofilm that appears to cause periodontal tissue breakdown may not be adequately represented within saliva.

There are also limitations of gut sampling. Most studies use fecal samples. These samples mostly represent distal luminal microbiota and may not represent mucosa-associated bacteria or small intestinal communities. This limitation should be explicitly acknowledged in future studies.

### 8.5. Prioritizing Disease-Specific Studies

Future research should not treat the oral–gut–systemic axis as a single pathway. The mechanisms may vary for different systemic diseases. Disease-specific studies are therefore needed.

IBD should be a priority because it is the most developed disease-specific model. Further research should integrate periodontal assessment, oral and gut microbiomes, IBD activity indices, medication data and inflammatory markers. Such studies should determine whether microbial signals linked to periodontitis add information beyond general oral–gut dysbiosis.

Oral–gut–liver models should be used to study both MASLD and NAFLD. Future human studies should assess tryptophan metabolites, AHR-related signatures, endotoxemia, hepatic inflammation and liver outcomes. Until stronger human data are available, RA, TMAO-related pathways and gastrointestinal cancer should be studied with caution.

### 8.6. Translational Priorities and Gut-Targeted Interventions

Currently, periodontal therapy remains the standard treatment for periodontitis. Interventions that impact the gut should be considered investigational. This includes probiotics, prebiotics, postbiotics, dietary fiber interventions, anti-inflammatory dietary patterns and metabolite-directed strategies [53,54].

Further studies can investigate combined periodontal and gut-directed approaches in well-defined populations, including patients with severe periodontitis and comorbid metabolic liver disease, obesity, autoimmune disease or chronic systemic inflammation.

Early studies should focus on biological endpoints, such as oral and gut microbiota, SCFA production, tryptophan metabolites, barrier markers, TMAO production and inflammatory mediators. Clinical outcome trials should follow only after reproducible biological effects are demonstrated.

The translational message should remain cautious. The axis may become useful for risk stratification, biomarker development and adjunctive therapy planning, but it is not ready for everyday clinical decision-making.

## 9. Conclusions

Periodontitis may serve as a continuous mucosal source of microbial, inflammatory and metabolic signals. These signals may reach the gut mainly through swallowed saliva and may influence gut microbial ecology, epithelial barrier function, mucosal immunity and systemic immune–metabolic pathways.

Current evidence supports the biological plausibility of the oral–gut–systemic axis in periodontitis. Human studies indicate associations, and experimental studies provide support for candidate mechanisms such as gut dysbiosis, barrier dysfunction, immune activation and microbial metabolite pathways.

However, the strength of evidence varies among disease models. IBD is currently the most developed model. Less mature axes include MASLD/NAFLD, rheumatoid arthritis, TMAO-related cardiometabolic pathways and gastrointestinal cancer, and they should be regarded as emerging or hypothesis-generating.

This field should now shift from general association to pathway-specific validation. Future studies should use longitudinal designs, strain-level source tracking, functional metagenomics, metabolomics and immune phenotyping. They should also apply standardized periodontal diagnosis and carefully control confounders.

Currently, periodontal therapy is the standard treatment for periodontitis. Probiotics, prebiotics, postbiotics, dietary interventions and metabolite-targeted strategies are investigational and should not be recommended as standard periodontal therapy.

## Figures and Tables

**Table 1:** Human evidence linking periodontitis with oral–gut microbiome disruption.

Study	Design/Material	Main Oral–Gut Finding	Interpretation/Main Limitation
Lourenco et al. [18]	Exploratory human study; stool microbiota in subjects with different periodontal status	Periodontal status was associated with differences in gut microbial profiles.	This was early human evidence. The study was exploratory and cannot prove causality.
Bao et al. [17]	Human paired saliva and fecal samples; severe periodontitis and controls	Severe periodontitis was associated with changes in both salivary and fecal microbiota.	The study supports saliva-mediated oral–gut microbial transfer. Directionality remains uncertain.
Lourenco et al. [19]	Human oral–gut bacterial profiling	Combined oral and gut bacterial profiles helped distinguish periodontal health from disease.	The study supports an oral–gut microbial signature of periodontitis. Clinical use is not yet established.
Baima et al. [15]	Human periodontal therapy study; oral and gut microbiota	Periodontal treatment was associated with changes in oral and gut microbial profiles.	The study suggests that periodontal therapy may influence the gut microbiome. Larger and longer studies are needed.
Miyauchi et al. [20]	Human gut microbiome and serum metabolome study	Patients with periodontitis showed persistent gut dysbiosis and a distinct serum metabolome.	The study links periodontitis with systemic metabolic changes. It does not define a direct causal pathway.
Baima et al. [16]	Longitudinal multi-omics study	Periodontitis and periodontal therapy were associated with taxonomic, functional and metabolic changes across oral and gut compartments.	This is important multi-omics evidence. It still requires validation in independent cohorts.
Luo, Xu and Yu et al. [44,45,46]	Mendelian randomization studies	These studies suggested possible causal links between gut microbiota and periodontitis risk.	The findings are hypothesis-generating. They do not identify direct microbial transmission or clinical mechanisms.
Yay et al. [47]	Oral and gut microbial profiling in periodontitis and Parkinson’s disease	Oral and gut microbial profiles were altered in a complex systemic disease context.	The study supports oral–gut microbial interaction, but disease-specific confounding is likely.

**Table 3:** Disease-specific molecular and immunological mechanisms within the oral–gut–systemic axis in periodontitis.

Disease/Systemic Axis	Main Oral–Gut Mechanism	Molecular or Immune Mediators	Key Evidence	Main Interpretational Limitation
Inflammatory bowel disease	Oral pathobiont transfer may disturb gut microbiota and worsen intestinal inflammation.	Gut dysbiosis, epithelial barrier dysfunction, inflammasome activation, Th17 cells, Th17/Treg imbalance, gut-homing T cells	Human studies, cohort data, systematic reviews, meta-analyses and experimental colitis/oral pathobiont mouse models [31,32,33,34,35,36,37,38,52]	This is the strongest disease-specific model, but causality and directionality are still not fully resolved.
MASLD/NAFLD	Periodontal dysbiosis may influence the gut–liver axis, especially in obesity or metabolic susceptibility.	Salivary microbiota transfer, gut dysbiosis, endotoxemia, tryptophan-AHR signaling, hepatic inflammatory and metabolic stress	High-fat diet or obesity-related mouse oral–gut–liver models and experimental periodontitis/salivary microbiota transfer studies [27,43]	Human validation is limited. Most evidence comes from animal models with obesity or high-fat diet exposure.
Rheumatoid arthritis	Periodontitis-related salivary microbiota may aggravate systemic immune dysregulation through the gut.	Gut dysbiosis, immune activation, inflammatory cell imbalance	Murine arthritis model using periodontitis-associated salivary microbiota [40]	The evidence is promising but early. Robust human oral–gut–RA data are still lacking.
Cardiometabolic/TMAO pathway	Periodontitis may alter gut microbial metabolism linked with TMAO production.	Gut microbial metabolism, TMAO, endothelial and metabolic inflammatory pathways	Preclinical periodontal treatment study in ApoE−/− mice [28]	This is a candidate pathway. It is not yet clear whether periodontal therapy changes cardiovascular risk through TMAO in humans.
Gastrointestinal cancer	Oral–gut microbial disruption may contribute to a pro-inflammatory or pro-tumor intestinal environment.	Oral pathobionts, gut dysbiosis, immune suppression, inflammatory signaling, colorectal cancer-related pathways	Narrative reviews and ligature-induced periodontitis mouse colorectal cancer model [39,41,42]	This remains hypothesis-generating. Cancer biology is multifactorial, and direct causal evidence is weak.

## Data Availability

No new data were created or analyzed in this study. Data sharing is not applicable to this article.

## Abbreviations

The following abbreviations are used in this manuscript:

AHR	aryl hydrocarbon receptor
ApoE−/−	apolipoprotein E-deficient
CD4	cluster of differentiation 4
CRC	colorectal cancer
CRP	C-reactive protein
DNA	deoxyribonucleic acid
GI	gastrointestinal
GPR109A	G protein-coupled receptor 109A
IBD	inflammatory bowel disease
IL-1β	interleukin-1 beta
IL-6	interleukin-6
IL-9	interleukin-9
IL-17	interleukin-17
LPS	lipopolysaccharide
MASLD	metabolic dysfunction-associated steatotic liver disease
MEDLINE	Medical Literature Analysis and Retrieval System Online
MMPs	matrix metalloproteinases
NAFLD	non-alcoholic fatty liver disease
OMVs	outer membrane vesicles
PRISMA	Preferred Reporting Items for Systematic Reviews and Meta-Analyses
RA	rheumatoid arthritis
SCFA	short-chain fatty acid
SCFAs	short-chain fatty acids
Th1	T helper 1
Th17	T helper 17
TMAO	trimethylamine N-oxide
TNF-α	tumor necrosis factor-alpha
Treg	regulatory T cell
